# Perioperative Artificial Intelligence Driven Integrated Modeling of Surgeries using Anesthetic, Physical and Cognitive Statuses for Predicting Hospital Outcomes

**DOI:** 10.21203/rs.3.rs-8000504/v1

**Published:** 2025-11-21

**Authors:** Sabyasachi Bandyopadhyay, Jiaqing Zhang, Ronald L. Ison, Bhanu Prasad Cherukuvada, David J. Libon, Patrick Tighe, Parisa Rashidi, Catherine Price

**Affiliations:** Stanford University; University of Florida; University of Florida; University of Florida; Rowan University; University of Florida; University of Florida; University of Florida

**Keywords:** preoperative status, clock drawing test, semi-supervised deep learning, interpretability

## Abstract

The association between preoperative cognitive status and surgical outcomes is a critical yet scarcely explored area. We assessed how preoperative cognitive status, as measured by clock drawing tests, contributed to predicting length of hospital stay, charges, pain during follow-up, and 1-year mortality beyond intraoperative variables, demographics, physical status, and comorbidities. We expanded our analysis to 6 surgical groups where sufficient data was available. Clock drawings were represented by 10 constructional features discovered by a semi-supervised deep learning algorithm, validated to differentiate between dementia and non-dementia patients. Machine learning models were trained using 5-fold cross-validation to classify postoperative outcomes. Shapley Additive Explanations analysis was used to find the most predictive features. Our results showed that the perioperative cognitive dataset served as the best dataset for 12 of 18 possible surgery-outcome combinations. Interpretability analysis showed that surgery duration was the most significant predictor of adverse outcomes, followed by anesthetic concentration. Disruptions in baseline correlations between intraoperative variables revealed that low average blood pressure and high standard deviation of blood pressure predicted adverse outcomes. Among the clock features, clock size was the most significant predictor of adverse outcomes. Our findings have relevance for improving healthcare modeling and perioperative risk prediction.

## INTRODUCTION

^1^Postoperative complications depend on preoperative health modulated by the surgery type and quality of anesthesia.^2^ Subjective measures of comorbidity, such as the American Society of Anesthesiologists (ASA) physical status score, are the most commonly used metrics for assessing preoperative health risk.^3^ However, such assessments may not be sufficient for predicting postoperative outcomes in older adults.^4,5^ Machine learning (ML) models and other heuristic scores have been used to stratify mortality risk and other specific outcomes using a combination of preoperative and intraoperative indices.^6–13^ However, studies that explored the relationships between pre-anesthesia physical status and intraoperative variables were generally undertaken for a specific surgery, except for a few studies^9,13^ where the risk of multiple adverse outcomes was stratified over a wide variety of surgery types. No study has yet investigated the impact of preoperative screeners beyond comorbidities for predicting postoperative outcomes.

Postoperative outcome prediction models built on intraoperative variables may be improved by examining the additional contribution of preoperative cognitive screeners beyond comorbidities. Preoperative cognitive screeners have been shown to predict the risk of postoperative delirium and mortality in older adults.^14–15,16^ However, these studies did not examine the predictive value of preoperative cognitive screener performance *in addition to* intraoperative variables (e.g., anesthesia type, depth, duration of surgery). Without this exploration, the added value of cognitive screeners cannot be fully assessed. This topic has become increasingly relevant due to the increasing number of older adults nationally,^17^ combined with the facts that older adult patients represent at least 50% of all surgeries^18^ and the expected rate of dementia is increasing on average 6% across the United States.^19^

**Figure 1:**
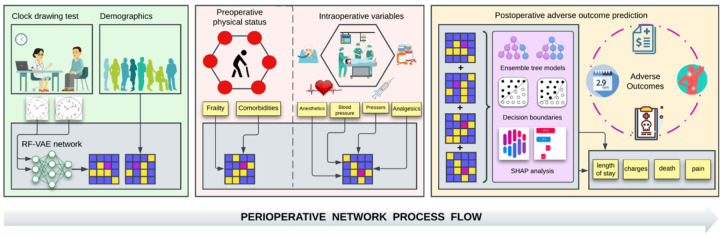
Perioperative Network Process Flow. Cognitive screeners such as the clock drawing test and demographic variables are collected preoperatively. Frailty and comorbidities are ascertained before surgery. Anesthetic, analgesic, and hemodynamic variables are collected during the operation. All data are combined into ensemble-based models to ascertain the postoperative incidence of adverse outcomes such as longer length of stay, higher hospital charges, higher pain, and mortality. Abbreviations: RF-VAE, relevance-factor variational autoencoder; SHAP, Shapley Additive Explanations.

For this investigation, we assessed the additive effect of preoperative cognitive screening performance as measured by the command and copy clock drawing test (CDT) over and above intraoperative variables, preoperative physical status, demographics, and comorbidities on postoperative outcomes for older adults electing surgeries (the workflow is shown in Fig. 1). Accurate clock construction to command and copy conditions assesses several cognitive abilities; differences in command versus copy conditions help differentiate types of cognitive deficits.^20^ We represented clock drawings using a semi-supervised deep learning (DL)-based 10-dimensional representation previously validated to capture unique constructional features of the CDT relevant to dementia.^21^ We hypothesized that irrespective of the surgical context, preoperative cognition as encoded by this DL representation of clock drawings would inform the classification of postoperative outcomes that are explicitly unrelated to cognition.

## RESULTS

### Participants

Individuals were primarily White, college-educated, and over 65 years of age (Table 1).

### Statistical Analyses

Figure 3 shows the cross-correlation between the various anesthetic and surgical features. Correlation values were categorized as high if they were greater than 0.5, moderate between 0.3 and 0.5, and low between 0.1 and 0.3 according to Cohen's d effect size criteria. Duration of surgery had a high positive correlation with oral MME in cardiovascular (Figure 3D), urologic (Figure 3E), and gynecologic (Figure 3F) surgeries, as well as when considering all surgeries together (Figure 3A). In orthopedic (Figure 3B), neurologic (Figure 3C), and otolaryngologic (Figure 3G) surgeries, surgery duration had a moderate positive correlation with oral MME. Phenylephrine had a high positive correlation with average blood pressure and standard deviation of blood pressure in all cohorts (Figure 3). Average blood pressure had a moderate positive correlation with standard deviation of blood pressure in orthopedic (Figure 3B), neurologic (Figure 3C), urologic (Figure 3E), gynecologic (Figure 3F), and otolaryngologic (Figure 3G) surgeries. This correlation became low in cardiovascular surgeries (Figure 3D) and when all surgeries were considered together (Figure 3A). Ephedrine had a moderate negative correlation with average blood pressure in gynecologic (Figure 3F) and otolaryngologic (Figure 3G) surgeries. Additionally, iso-sev-MAC had a moderate negative correlation with propofol in orthopedic (Figure 3B), urologic (Figure 3E), gynecologic (Figure 3F), and otolaryngologic (Figure 3G) surgeries.

### Artificial Intelligence

#### Model Performances

The best ML models for each outcome and each surgical context are reported in Table 2, as is the best dataset for predicting hospital outcomes and its corresponding division of samples between classes 0 and 1. Table 3 highlights dataset predictive features. In 12 of 22 cases, the *Perioperative cognitive dataset* generated the best model; in 5 instances, the *Perioperative dataset* gave the best model, and in 5 other instances, the *Intraoperative dataset* gave the best model. Charges classification was performed superlatively across all surgeries, and LOS classification was performed moderately in orthopedics, urology, and gynecology surgeries. However, average pain during follow-up proved to be a complex classification problem. One-year mortality, which could only be classified for all surgeries, had very low precision and F1 score, indicating the prevalence of many false positives compared to true positives. This is partially due to the heavy class imbalance present in this dataset.

#### Interpretability analysis

SHAP analysis was performed on each best model to find the top 10 most informative features. Figure 4 shows the SHAP results for the different surgery groups.

In 16 of 18 SHAP plots, surgery duration was the most important predictor. In the other two plots (Figures 4C, 4I), it was the second most important predictor. Besides surgery duration, iso-sev-MAC is among the top 2 most predictive features in 7 of 18 situations. The baseline correlations between the surgical variables in each surgery are shown in Figure 3. The Results section details which associations were maintained and reversed in the SHAP plots. This indicates which correlations were broken in patients who experienced adverse outcomes (i.e., longer LOS, higher charges, and more average pain).

### Intraoperative Characteristics of Adverse Outcomes

#### Orthopedic Surgery

Figure 4A. Higher LOS was associated with high values of SD NIBP and low values of average deviation of noninvasive blood pressure (AVG NIBP) during surgery. This is opposite to the correlation expected from Figure 3B. The same pattern is further exaggerated in average pain, where AVG NIBP during surgery is the most important predictor, and SD NIBP is the third most important predictor of greater pain (Figure 4C).

#### Neurosurgery

Figure 4D. The model associated higher doses of ephedrine sulfate, high AVG NIBP, and high SD NIBP during surgery with longer LOS. This pattern is distinct from the correlations between these variables in Figure 3C. The same pattern is repeated in average pain classification (Figure 4F); additionally, the ephedrine sulfate dose is a more important predictor than the blood pressure values. Figure 4E reveals that longer durations of surgery, lower values of iso-sev-MAC (topmost features), and lower AVG NIBP but higher SD NIBP (less critical) were associated with higher hospital charges. These patterns are also in contrast with the correlations to be expected in the neurosurgery group from Figure 3C.

#### Cardiac and Vascular

Figure 4I. Patients who experienced a low SD NIBP while being exposed to high doses of phenylephrine hydrochloride (HCl) pressor were predicted to experience greater average pain (Figure 3D). Cardiovascular surgery patients should have high SD NIBP if they receive high volumes of phenylephrine. In comparison to the orthopedic and neurologic surgeries, LOS and charges in this surgery group were more amply predicted by preexisting comorbidities.

#### Urologic

Figure 4J through Figure 4L. Deviations from the baseline correlation patterns found in Figure 3E.

#### Gynecologic

Figure 4N. Hospital charges in gynecologic surgeries depend solely on and are driven by the surgery duration. Figure 3F. In the gynecologic surgical group, ephedrine sulfate negatively correlates with phenylephrine HCl and AVG NIBP. Figure 4O. Higher postoperative pain in this cohort was predicted by low phenylephrine HCl, low ephedrine sulfate, high SD NIBP (more critical), and high AVG NIBP.

#### Otolaryngologic

Figure 4P. People who experienced high SD NIBP while simultaneously showing low mean intraoperative blood pressure were predicted to have longer LOS. This is different from the positive correlation between mean NIBP and SD NIBP in this surgical group (Figure 3G). The same pattern is exaggerated in Figure 4R (average pain). Figure 4R shows that the incidence of higher iso-sev-MAC and higher propofol—two negatively correlated variables—predicted greater average pain during follow-up. Figure 4Q shows that in this group, the duration of surgery drives charges.

The loss of a positive association between AVG NIBP and SD NIBP and specifically higher SD NIBP with lower AVG NIBP predicted prolonged LOS and/or greater average pain in 3 of 6 surgical contexts (orthopedic, cardiovascular, and otolaryngological).

### Comorbidities and Disparities in Adverse Outcomes

#### Orthopedic

Figure 4A. Being older and frail was associated with longer LOS after orthopedic surgery. Figure 4B. Patients with higher education were predicted to pay fewer hospital charges. Figure 4C. Being older was associated with reporting less average pain. Also, higher frailty and hypertension were associated by the best model with higher average pain.

#### Neurosurgery

Figure 4E. In neurosurgery, White patients were predicted to pay less hospital charges than non-White patients. In comparison, patients with hyperlipidemia and movement disorders were predicted to pay higher charges than others.

#### Cardiovascular

Figure 4G. Hyperlipidemia, hypertension, and sleep apnea predicted longer LOS, while diabetes predicted lower LOS. The model associated being biologically female and/or Black with lower LOS. Figure 4H. Higher education, hyperlipidemia, and hypertension predicted higher charges.

#### Urologic

Figure 4J. Higher education predicted lower LOS; also, hyperlipidemia and diabetes predicted lower LOS in urologic surgeries. Figure 4K. Diabetes also predicted lower charges for this surgery group. Figure 4L. Older age predicted lower self-reported pain in this surgery group.

#### Gynecologic

Figure 4M. Higher education and higher ASA scores predicted greater LOS. Hyperlipidemia predicted higher LOS, but hypertension predicted lower LOS in this group. Figure 4O. Older age predicted lower reported pain, and higher ADI predicted greater pain in gynecologic surgeries.

#### Otolaryngologic

Comorbidities or demographic disparities did not strongly predict adverse outcomes in otolaryngologic surgeries.

Older people were predicted to report less pain in 3 surgical contexts: orthopedic, urologic, and gynecologic. Hyperlipidemia predicted longer LOS in 2 surgical contexts: gynecologic and cardiovascular. Hyperlipidemia also predicted higher hospital charges in 2 surgeries: neurologic and cardiovascular.

### Clock Drawing Characteristics

Preoperative clock drawing characteristics were important predictors in 9 of 18 surgery-outcome combinations. Table 3. Among these, clock size was the most critical cognitive predictor in five surgery-outcome contexts: two orthopedic (LOS, charges), one neurosurgery (charges), one cardiovascular (charges), one gynecology (LOS). Figures 4A, 4B, 4E, 4H, 4M. In *orthopedic surgery*, a larger clock size predicted greater LOS and greater hospital charges. Figures 4A, 4B. In contrast, a larger clock predicted lower charges and lower LOS in neurosurgery, cardiovascular surgery, and gynecologic surgery, respectively. Figures 4E, 4H, 4M. Clock size was also a significant predictor in two of the remaining four surgery-outcome combinations (urology-charges, and otolaryngology-LOS). Figures 4K, 4P. The rotated ellipse and/or rotated vertical ellipse predicted longer LOS in *orthopedic surgery* (Figure 4A), lower hospital charges in *neurosurgery* (Figure 4E), lower hospital charges in *cardiovascular surgery* (Figure 4H), higher pain in *urologic surgery* (Figure 4L), and lower LOS in *gynecologic surgery* (Figure 4M). It was also a significant predictor in otolaryngology-LOS, but the directionality could not be easily understood (Figure 4P). Upward displaced clock hands predicted lower charges in *orthopedic surgery* (Figure 4B), lower LOS, higher charges, and higher pain in *urologic surgery* (Figures 4J, 4K), and higher LOS in *otolaryngology surgery* (Figure 4P). Obovate clocks predicted higher charges in *orthopedic surgery* (Figure 4B), higher LOS and lower pain in *urologic surgery* (Figures 4J, 4L), and lower LOS in *gynecology surgery* (Figure 4M). An obtuse angle between hands predicted lower charges in *orthopedic surgery* (Figure 4B), *cardiovascular surgery* (Figure 4H), and higher LOS in *urologic surgery* (Figure 4J). Therefore, after clock size, two clock shape features (elliptical and obovate) and two hand placement features (upward displacement and wide angle between) were the most significant.

## DISCUSSION

This study demonstrates an integrated artificial intelligence–driven modeling of surgery outcomes. A complex array of cognitive, anesthetic, and physical variables predicted longer length of stay, higher hospital charges, higher average pain, and mortality. Six different surgery types were individually studied, including *orthopedic*, *neurologic*, *cardiovascular*, *urologic*, *gynecologic*, and *otolaryngologic* surgeries. Machine learning classifiers were developed in each case to maximize recognition of adverse outcomes. We interpreted the models to understand how perioperative variables impacted the degree and directionality of influence.

### Intraoperative Networks

The cross-correlation patterns of the intraoperative variables show significant positive associations between surgery duration, OME, and iso-sev-MAC, and a secondary group of strong positive associations between AVG NIBP, SD NIBP, and phenylephrine. Phenylephrine HCl and ephedrine sulfate, both vasopressors, were negatively correlated. The first group of strong positive correlations represents the *anesthetic and analgesic network*. The second group represents the *hemodynamic management network*. These represent the two primary housekeeping tasks during any surgical procedure.

Surgical contexts, such as orthopedic, urologic, gynecologic, and otolaryngologic surgeries, exhibited a robust negative association between propofol and iso-sev-MAC. However, in patients who experienced adverse outcomes, the inter-variable associations diverged from the correlation networks (Supplementary Figure 3). The two correlation networks showed the expected correlation patterns between the surgical variables. At the same time, the relative directionality of these features found in the SHAP analysis plots from ML models characterized the patients who experienced postoperative adverse outcomes. In adverse outcomes, the variables in the *hemodynamic network* diverged more from the expected correlation patterns than the *anesthetic and analgesic network* variables. The *hemodynamic network* showed a deviation from expected correlation 9 of 12 times, while the *anesthetic network* did so 2 of 12 times when their features were significant predictors of adverse outcomes in the ML models. This observation, combined with the fact that the most common disruption in the *hemodynamic network* was higher variance in blood pressure with low AVG NIBP, indicates that blood loss during surgery was a driver of postoperative adverse outcomes. Despite not being disrupted as much as the *hemodynamic network*, the *anesthetic and analgesic network* was the more important of the two in predicting adverse outcomes because duration of surgery was the most significant predictor in our study. Furthermore, iso-sev-MAC was within the top 2 most relevant predictors in 7 of 18 scenarios, while oral MME was within the top 10 predictors in 7 of 18 scenarios, suggesting that a combination of longer surgery and greater depth of anesthesia and analgesia concertedly generated adverse postoperative effects.

### Significance of Clock Features

The CDT represented a preoperative cognition “snapshot.” The CDT to command and copy condition was projected onto a pretrained RF-VAE, DL encoder, representing clock drawing in a combination of 10 unique, mutually disentangled constructional features of the clockface.^21^ These features had been previously validated to distinguish between clocks drawn by dementia and non-dementia patients. Among the features investigated, clock size was the most important predictor. Next in importance was a distorted clock shape represented by rotated ellipticity, obovateness, and abnormal time representation as seen by the upward displacement and obtuse angle between the clock hands. Successful clock drawing requires the coordination of multiple neurocognitive operations, including access to semantic information, mental planning, visuospatial reasoning, and motor ability.^22^ In a community-dwelling, non-dementia sample, clock drawing has been linked to the integrity of MRI white matter connections^23^ and neuropsychological test parameters,^24^ further illustrating that multiple neurocognitive operations must be coordinated for successful test performance. Clock size and shape aberrations are frequently present in patients with dysexecutive impairment.^25–27^ Incorrect time representation (i.e., incorrectly drawing the clock hands) is associated with semantic and dysexecutive deficits.^22^ The digital clock drawing test provided data not otherwise available in hospital records.

### Importance of Variable Groups

Although the perioperative cognitive variables demonstrated the strongest classification performance in 12 of 18 surgery-outcome combinations, the difference in performance from the other variable sets, especially the intraoperative variables, was not considerable. This indicated that intraoperative variables were the strongest predictors of postsurgical complications. However, in many instances the addition of clock features either improved the specificity or improved sensitivity-specificity balance; for example, (A) LOS, charges, and mortality in all surgeries; (B) charges in *orthopedic* surgery; (C) LOS and charges in *neurosurgery*; (D) LOS in *urologic* surgery; and (E) LOS in *gynecologic* surgery. This suggests preoperative cognition had an impact on postoperative recovery in multiple surgical contexts.

### Strengths

Our study has unique strengths. First, the study applied ML to a wide array of data points from preoperative and intraoperative variables to classify postoperative outcomes. Herein, we performed an integrated assessment of different preoperative datasets, including cognitive features for predicting postsurgical outcomes. ML classifiers with different inductive biases were used to improve the generality of classification performance over the different datasets for the diverse surgery outcome combinations. The application of ML models allowed the examination of nested, nonlinear relationships within the multimodal datasets. Interpretability analysis using SHAP helped us understand the characteristics of adverse outcomes from the lenses of intraoperative, preoperative physical status, and cognitive variables. This led to an improved understanding of perioperative variables' contribution to postoperative outcomes. Future models may help improve healthcare modeling for cost reduction, rate of readmissions, and post discharge health management.

### Limitations

The size of the initial dataset was significantly impacted by missing entries. The final dataset used for classification was one-fourth of the size of the initial dataset. Our study was limited by the binary classification setting. However, the use of binary classification allowed us to test diverse surgeries within the same framework. In the future, individual surgeries should be studied with appropriate postoperative outcomes, and DL-based clustering or self-supervised learning can be used to learn compressed representations of the surgeries themselves. These ideas, combined with multitask learning, could generate a deep surgical signature for downstream classification of postoperative complications. Transfer learning can create information that can be translated to other datasets/tasks. We will also explore this in future works.

## CONCLUSION

This paper demonstrates an integrated AI-driven modeling of a diverse group of surgical procedures for classifying multiple postoperative outcomes. A perioperative network of variables encompassing preoperative cognitive factors provides predictive value beyond intraoperative variables. It also emphasizes the importance of intraoperative anesthetic, analgesic, and hemodynamic factors. Hemodynamic mismanagement and increased surgery duration, anesthesia depth, and analgesia dosage all contributed significantly to the development of postoperative adverse effects. Findings have relevance for improving healthcare modeling.

## METHODS

### Participants

Data were collected via a federally funded investigation approved through the UF Institutional Review Board and involving a Health Insurance Portability and Accountability Act (HIPAA) waiver, consent, and data extraction from electronic medical records through an honest data broker. The study followed Declaration of Helsinki and respective university guidelines, and TRIPOD criteria.

The original surgical dataset comprised 22,473 patients aged 65 years or older who completed an in-person preoperative assessment for elective surgeries between January 2018 and December 2019. Participants were excluded if non-fluent in English, had <4 years of education, or had some visual, motor, or hearing impairment severely limiting their ability to execute a CDT.^28,20,29–32^ From these data, we possessed complete intraoperative data for 18,037 patients, preoperative physical status information for 12,422 patients, complete demographics (age, race, sex, ethnicity, education, and area deprivation index [ADI]^33^) for 10,055 patients, and clock drawings for 11,777 patients. Supplementary Figure 1 shows an integrated consensus diagram illustrating the different data modalities used for creating the datasets reported in this study. A stepwise combination of these data-modalities was carried out to develop the three datasets primarily used for our analysis. These three datasets were divided according to surgery types (Supplementary Figure 1) to create surgery-specific intraoperative, perioperative, and perioperative cognition datasets used for analysis.

### Categories of Variables

The primary variables are within 4 categories:

- *Intraoperative variables*: type of surgery, surgery duration (minutes), propofol dose (mg), oral morphine milligram equivalent (MME) dose (mg), anesthetic depth as represented by the mean alveolar concentration of isoflurane or sevoflurane (iso-sev-MAC), mean and standard deviation of noninvasive blood pressure (SD NIBP), and doses of the vasopressors phenylephrine (mcg) and ephedrine (mg).
- *Demographics*: age, sex, race, ethnicity, years of education, and ADI (from location of residence). For analysis consisting of all surgeries, the surgery type was one-hot encoded. Surgery type was defined as the patient’s primary surgical service.
- *Preoperative physical status*: ASA physical status score,^34^ body mass index (BMI) at admission, frailty,^35^ and comorbidities. Admission BMI was not used in the final analyses because greater than 60% of the data on this variable were missing. Comorbidities present in our dataset included sleep apnea, diabetes, hyperlipidemia, hypertension, movement disorders, and cognitive disorders.
- *Preoperative cognitive screener*: Every participant completed a preoperative clock drawing to command and copy conditions as part of a hospital-wide preoperative screening program for older adults.^36^ Per published instructions, individuals were required to “draw the face of a clock, put in the numbers, and set the hands to ten after eleven” and then copy a model clock.^26^ Clock drawing to command condition requires the simultaneous syntactic comprehension of verbal instructions, recalling the semantic attributes of a clock, working memory, accurate mental planning, and fine motor skills.^37^ In the copy condition, clock construction relies on visual scanning abilities, visuoconstruction, and executive functioning.^22^ Clock drawings were captured using digital pen technology.^32^ Final output images of clock drawings to command and copy conditions were projected on a 10D DL latent space described in our previous work.^21,28^ The deep CDT representation in this study originated from a latent representation developed using semi-supervised training of a relevance-factor variational autoencoder (RF-VAE) network to classify dementia from non-dementia participants.^21^ This RF-VAE network was trained using 23,521 unlabeled clock drawings collected as part of a routine check-up in the preoperative setting.^36,38^ The RF-VAE discovered 10 unique constructional aspects of the CDT that were validated to be different in people with dementia versus healthy peers using a smaller classification dataset. RF-VAE–trained network clocks overlapped with the current study. Supplementary Figure 3A-B shows the 10 unique constructional features described in the above study. Supplementary Figure 3C shows their relative occurrence in dementia samples. All numerical variables were scaled to their respective maximum values to create a normalized dataset, and all categorical variables were one-hot encoded. Six individual surgeries contained sufficient data for downstream analyses: orthopedics, neurosurgeries, cardiac and vascular surgeries, urologic surgeries, gynecologic surgeries, and otolaryngologic surgeries.

### Outcomes

Postoperative outcomes of interest were (A) *length of hospital stay* (LOS; hours), (B) *hospital charges* (dollars), (C) *1-year mortality*, and (D) *average pain* during the follow-up period. Mortality classification was not performed for individual surgeries due to the low number of deaths falling in each cross-validation fold and in the hold-out test dataset. Outcomes were binarized:

- LOS = 0: same-day discharge, 1: longer than one day,
- Hospital charges = 0: less than $30,000, 1: greater than or equal to $30,000
- Mortality = 0: survived until one year after discharge, 1: died within one year of being discharged
- Average pain = 0: average pain = 0, 1: average pain ≥ 1. The average pain rating was calculated by averaging the pain score reported by patients according to the Brief Pain Inventory Short Form.^39^ It was reported on a scale of 0 to 10. The same thresholds were used for individual surgeries.

### Cohorts

**Figure 2:**
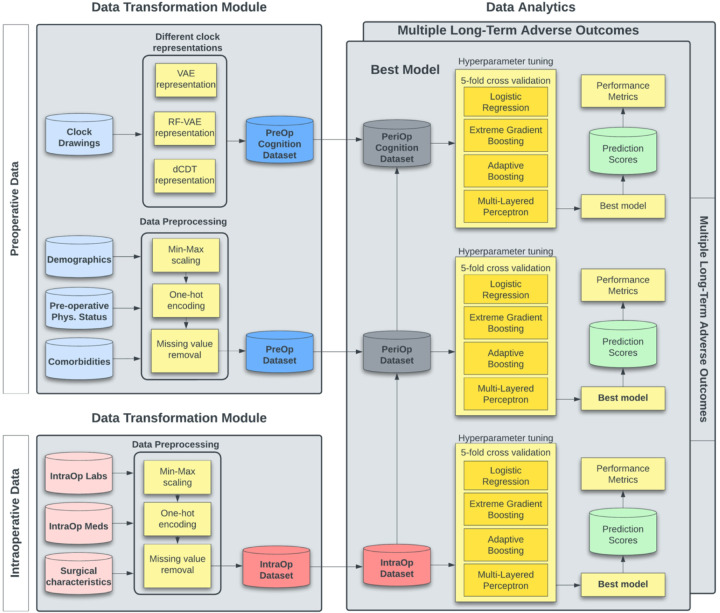
Perioperative AI-Based Integrated Modeling of Surgeries (Peri-AIIMS) Framework. Intraoperative variables are converted into one-hot encoded vectors or min-max scaled numeric vectors. Cognitive status represented by clock drawings are projected onto the 10D latent RF-VAE latent space as described by Bandyopadhyay et al, 2023^25^. Demographics and comorbidities are one-hot encoded. Preoperative physical status variables (frailty and ASA) are min-max scaled. Multiple models are trained within 5-fold cross-validation using either the intraoperative, perioperative, or perioperative cognition datasets. In each case, the best model for each adverse outcome is reported in the paper. Abbreviations: RF-VAE, relevance-factor variational autoencoder.

**Figure 3:**
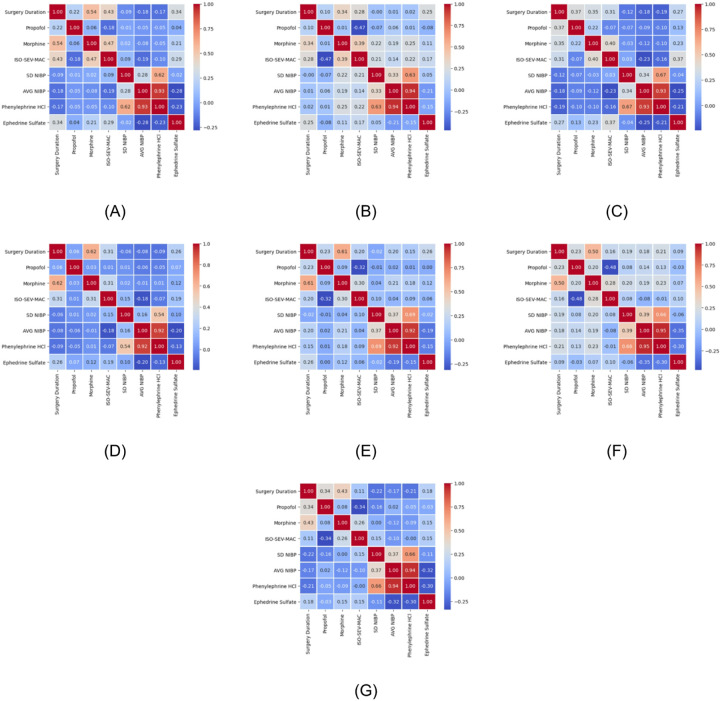
Cross-Correlation Matrix Between the Different Intraoperative Features of Surgeries. (A) All surgeries, (B) Orthopedic surgeries, (C) Neurosurgeries, (D) Cardiovascular surgeries, (E) Urologic surgeries, (F) Gynecologic surgeries, and (G) Otolaryngologic surgeries. Abbreviations: AVG NIBP, average deviation of noninvasive blood pressure; HCl, hydrochloride; iso-sev-MAC, mean alveolar concentration of isoflurane and sevoflurane; SD NIBP, standard deviation of noninvasive blood pressure.

**Figure 4:**
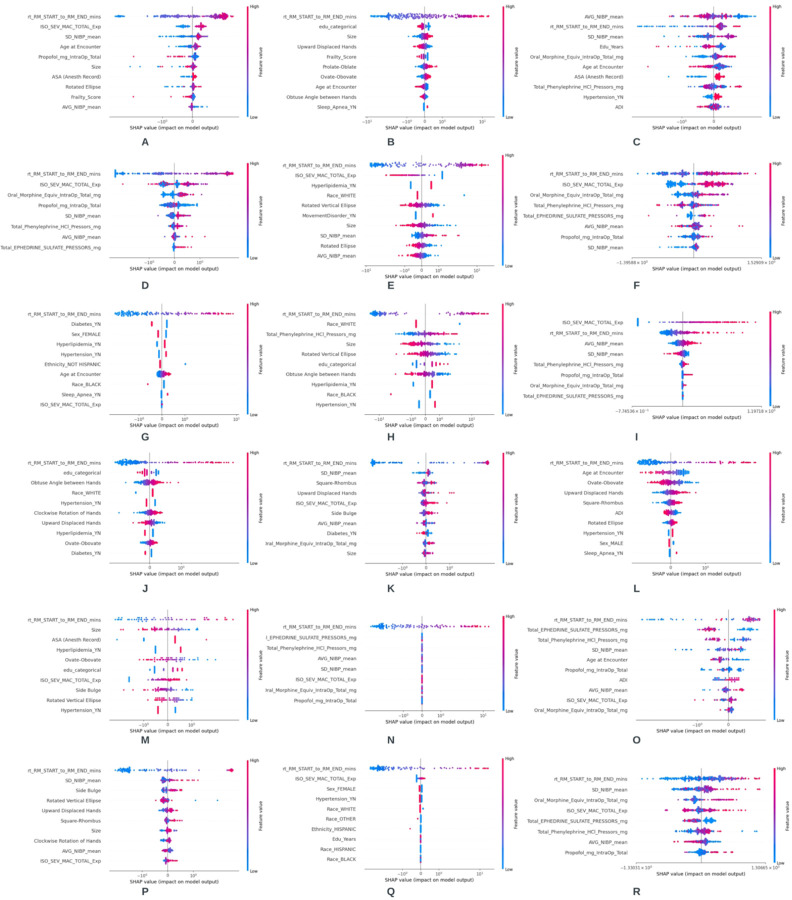
SHAP Interpretation Plots for Best Models Showing Top 10 Features. (A) Orthopedic surgery LOS; (B) Orthopedic surgery charges; (C) Orthopedic surgery average pain; (D) Neurosurgery LOS; (E) Neurosurgery charges; (F) Neurosurgery average pain; (G) Cardiac and vascular surgery LOS; (H) Cardiac and vascular surgery charges; (I) Cardiac and vascular surgery average pain; (J) Urologic surgery LOS; (K) Urologic surgery charges; (L) Urologic surgery average pain; (M) Gynecology surgery LOS; (N) Gynecology surgery charges; (O) Gynecology surgery average pain; (P) Otolaryngology surgery LOS; (Q) Otolaryngology surgery charges; (R) Otolaryngology surgery average pain. Abbreviations: LOS, length of hospital stay.

Three different feature combinations evaluated the potential additive effect of data modalities on classification: (A) *intraoperative dataset* from anesthetic, blood pressure, and surgical features; (B) *perioperative dataset* combined the *intraoperative dataset* with demographics, preoperative physical status, and comorbidities; and (C) *perioperative cognitive dataset*, combined the *perioperative dataset* with DL representation of clock drawings (Figure 2; Supplementary Figure 1). Command clock drawings and copy clock drawings were tested separately and in concert. The best performance among these three datasets was reported for each classification task.

### Procedure

#### Statistical Analysis

Pearson's product-moment correlation matrices were calculated for every surgery type between the different intraoperative variables. Cohen's d effect sizes were used to evaluate the correlation values' significance level.

#### Artificial Intelligence

An array of ML models, including logistic regression (LR), random forest, naïve Bayes classifier, extreme gradient boosting (XGBoost),^40^ adaptive boosting (AdaBoost),^41^ categorical boosting (CatBoost),^42^ and a multilayered perceptron (MLP)^43^ were trained on the training subset of each dataset in different surgical contexts. The hyperparameters of these models were optimized inside a 5-fold cross-validation setting. Area under the receiver operating curve (AUROC), accuracy, F1 score, sensitivity, specificity, and average precision were reported on the test dataset. The best model was selected based on the performance, least time complexity, and ease of interpretability. Shapley Additive Explanations (SHAP) analysis was performed for the best model. The test dataset was bootstrapped 100 times with replacement to generate 95% confidence intervals.

## Supplementary Material

Supplementary Files

This is a list of supplementary files associated with this preprint. Click to download.

- supplementaryScientificReport.docx
- SupplementaryLegends.docx

## Figures and Tables

**Table 1. -:** Patient Demographics and Comorbidities

	All Surgeries	Orthopedics	Neurology	Cardiac and Vascular	Urology	Gynecology	Otolaryngology
N	6221	916	542	461	639	203	395
Age (years)	73.3 ± 6.0	72.0 ± 5.6	73.0 ± 5.3	73.9 ± 6.1	74.1 ± 6.2	72.5 ± 5.7	73.9 ± 6.3
Sex (Male, Female) (%)	50.3, 49.7	44.3, 55.7	55.1, 44.8	49.9, 50.1	71.0, 28.9	0.0, 100.0	54.9, 45.1
Race (White, Black) (%)	87.0, 7.3	86.6, 6.0	93.3, 2.7	86.9, 8.2	87.5, 6.7	88.7, 7.4	88.3, 5.3
Ethnicity (non-Hispanic, Hispanic) (%)	95.7, 2.0	93.8, 1.5	97.2, 2.0	94.3, 2.8	97.3, 2.0	98.0, 1.9	96.2, 1.8
ADI	60.2 ± 23.0	59.1 ± 23.0	54.7 ± 24.1	60.9 ± 21.9	61.0 ± 22.7	62.5 ± 21.9	58.7 ± 22.3
Education (years)	13.9 ± 2.9	14.4 ± 3.1	14.3 ± 2.8	13.4 ± 2.7	14.2 ± 3.1	13.8 ± 2.4	13.8 ± 2.8
ASA (%)	1: 0.1, 2: 11.5, 3: 79.5, 4: 8.9, 5: 0.01	1: 0.0, 2: 17.5, 3: 80.2, 4: 2.3, 5: 0.0	1: 0.4, 2: 10.7, 3: 86.7, 4: 2.2, 5: 0.0	1: 0.0, 2: 1.5, 3: 70.5, 4: 28.0, 5: 0.0	1: 0.0, 2: 13.1, 3: 84.2, 4: 2.7, 5: 0.0	1: 0.5, 2: 25.1, 3: 71.9, 4: 2.5, 5: 0.0	1: 0.0, 2: 14.9, 3: 80.0, 4: 5.1, 5: 0.0
Frailty	1.2 ± 1.3	1.4 ± 1.3	1.2 ± 1.4	1.3 ± 1.4	1.0 ± 1.3	1.1 ± 1.2	1.0 ± 1.3
Comorbidities (%)	SA: 16.0, DB: 29.4, HL: 54.4, HT: 55.1, MD: 5.8, CD: 0.9	SA: 16.5, DB: 23.8, HL: 57.2, HT: 59.9, MD: 4.6, CD: 0.9	SA: 15.5, DB: 25.6, HL: 52.0, HT: 55.1, MD: 27.5, CD: 1.1	SA: 12.1, DB: 33.8, HL: 64.4, HT: 41.4, MD: 2.1, CD: 0.4	SA: 13.8, DB: 28.5, HL: 43.9, HT: 54.3, MD: 3.7, CD: 0.9	SA: 8.4, DB: 20.7, HL: 43.3, HT: 52.7, MD: 1.9, CD: 0.5	SA: 16.2, DB: 29.6, HL: 40.7, HT: 57.9, MD: 3.0, CD: 1.0

Abbreviations: ADI, area deprivation index; ASA, American Society of Anesthesiologists Score; CD, cognitive disorder; DB, diabetes; HL, hyperlipidemia; HT, hypertension; MD, movement disorder; SA, sleep apnea.

**Table 2. -:** Performance of Machine Learning Models on Long-Term Postsurgical Outcomes Over Different Surgeries

Surgery Type	Outcome	Best Dataset (N_0_, N_1_)	Best Model	AUC (95% CI)	Accuracy (95% CI)	F1 Score (95% CI)	Precision (95% CI)	Sensitivity (95% CI)	Specificity (95% CI)
**All surgeries**	LOS	Peri-Op Cognitive (0: 3053, 1: 1908)	XGBoost	0.93 (0.92 – 0.95)	0.87 (0.85 – 0.89)	0.83 (0.81 – 0.85)	0.82 (0.79 – 0.84)	0.85 (0.82 – 0.88)	0.88 (0.86 – 0.90)
	Charges	Peri-Op Cognitive (0: 2341, 1: 2605)	XGBoost	0.98 (0.97 – 0.99)	0.93 (0.92 – 0.94)	0.93 (0.92 – 0.95)	0.94 (0.93 – 0.96)	0.92 (0.91 – 0.94)	0.94 (0.92 – 0.95)
	Average Pain	Peri-Op (0: 2309, 1: 3084)	LR	0.82 (0.80 – 0.84)	0.77 (0.75 – 0.79)	0.80 (0.78 – 0.82)	0.80 (0.77 – 0.83)	0.80 (0.77 – 0.82)	0.73 (0.70 – 0.76)
	1-year Mortality	Peri-Op Cognitive (0: 4740, 1: 226)	LR	0.71 (0.66 – 0.77)	0.70 (0.68 – 0.73)	0.16 (0.11 – 0.20)	0.09 (0.06 – 0.12)	0.61 (0.50 – 0.73)	0.71 (0.69 – 0.73)
**Orthopedics**	LOS	Peri-Op Cognitive (0: 207, 1: 662)	XGBoost	0.84 (0.79 – 0.88)	0.83 (0.79 – 0.88)	0.90 (0.87 – 0.93)	0.85 (0.82 – 0.89)	0.95 (0.92 – 0.97)	0.47 (0.35 – 0.58)
	Charges	Peri-Op Cognitive (0: 82, 1: 787)	LR	0.99 (0.99 – 1.00)	0.95 (0.92 – 0.97)	0.97 (0.96 – 0.98)	1.00 (1.00 – 1.00)	0.95 (0.91 – 0.97)	1.00 (1.00 – 1.00)
	Average Pain	Peri-Op (0: 156, 1: 752)	XGBoost	0.69 (0.62 – 0.77)	0.84 (0.80 – 0.88)	0.91 (0.88 – 0.93)	0.86 (0.82 – 0.89)	0.97 (0.95 – 0.98)	0.26 (0.16 – 0.38)
**Neurology**	LOS	Intra-Op (0: 430, 1: 693)	XGBoost	0.92 (0.88 – 0.94)	0.86 (0.82 – 0.90)	0.89 (0.86 – 0.92)	0.87 (0.83 – 0.91)	0.91 (0.87 – 0.94)	0.79 (0.72 – 0.84)
	Charges	Peri-Op Cognitive (0: 90, 1: 411)	LR	0.93 (0.87 – 0.97)	0.86 (0.80 – 0.91)	0.91 (0.87 – 0.94)	0.94 (0.89 – 0.98)	0.88 (0.82 – 0.92)	0.76 (0.60 – 0.91)
	Average Pain	Intra-Op (0: 328, 1: 795)	XGBoost	0.74 (0.68 – 0.80)	0.78 (0.74 – 0.82)	0.86 (0.83 – 0.88)	0.81 (0.77 – 0.86)	0.90 (0.87 – 0.93)	0.49 (0.41 – 0.58)
**Cardiac and vascular**	LOS	Peri-Op (0:192, 1: 260)	LR	0.89 (0.83 – 0.93)	0.83 (0.78 – 0.88)	0.85 (0.80 – 0.90)	0.86 (0.79 – 0.94)	0.84 (0.76 – 0.92)	0.82 (0.73 – 0.91)
	Charges	Peri-Op Cognitive (0: 93, 1: 359)	LR	0.96 (0.93 – 0.98)	0.91 (0.85 – 0.95)	0.94 (0.90 – 0.97)	0.98 (0.95 – 1.00)	0.90 (0.82 – 0.95)	0.95 (0.82 – 1.00)
	Average Pain	Intra-Op (0: 307, 1: 521)	LR	0.70 (0.64 – 0.76)	0.64 (0.59 – 0.70)	0.69 (0.64 – 0.75)	0.76 (0.70 – 0.83)	0.63 (0.55 – 0.70)	0.66 (0.56 – 0.74)
**Urology**	LOS	Peri-Op Cognitive (0: 383, 1: 148)	LR	0.80 (0.71 – 0.87)	0.77 (0.71 – 0.82)	0.63 (0.52 – 0.72)	0.57 (0.44 – 0.69)	0.70 (0.59 – 0.81)	0.80 (0.74 – 0.86)
	Charges	Peri-Op Cognitive (0: 295, 1: 236)	XGBoost	0.96 (0.93 – 0.99)	0.93 (0.89 – 0.96)	0.91 (0.86 – 0.95)	0.94 (0.88 – 1.00)	0.88 (0.81 – 0.94)	0.96 (0.92 – 1.00)
	Average Pain	Peri-Op Cognitive (0: 294, 1: 237)	LR	0.75 (0.69 – 0.81)	0.70 (0.64 – 0.76)	0.70 (0.64 – 0.76)	0.78 (0.68 – 0.86)	0.64 (0.55 – 0.72)	0.78 (0.69 – 0.88)
**Gynecology**	LOS	Peri-Op Cognitive (0: 123, 1: 50)	LR	0.80 (0.67 – 0.89)	0.72 (0.62 – 0.83)	0.56 (0.33 – 0.71)	0.52 (0.29 – 0.72)	0.60 (0.35 – 0.81)	0.78 (0.64 – 0.89)
	Charges	Intra-Op (0: 254, 1: 128)	LR	0.99 (0.97 – 1.00)	0.94 (0.89 – 0.97)	0.95 (0.91 – 0.98)	1.00 (1.00 – 1.00)	0.91 (0.84 – 0.96)	1.00 (1.00 – 1.00)
	Average Pain	Peri-Op (0: 167, 1: 33)	XGBoost	0.74 (0.62 – 0.88)	0.79 (0.70 – 0.88)	0.87 (0.82 – 0.93)	0.87 (0.80 – 0.95)	0.88 (0.79 – 0.94)	0.36 (0.12 – 0.68)
**Otolaryngology**	LOS	Peri-Op Cognitive (0: 228, 1: 111)	XGBoost	0.88 (0.80 – 0.94)	0.82 (0.75 – 0.88)	0.69 (0.52 – 0.78)	0.86 (0.71 – 0.95)	0.58 (0.41 – 0.71)	0.95 (0.90 – 0.99)
	Charges	Peri-Op (0: 243, 1: 137)	LR	0.95 (0.91 – 0.98)	0.87 (0.80 – 0.92)	0.88 (0.82 – 0.93)	0.97 (0.91 – 1.00)	0.81 (0.72 – 0.90)	0.96 (0.87 – 1.00)
	Average Pain	Intra-Op (0: 580, 1: 238)	XGBoost	0.63 (0.54 – 0.70)	0.71 (0.65 – 0.75)	0.81 (0.76 – 0.84)	0.76 (0.70 – 0.81)	0.86 (0.81 – 0.91)	0.33 (0.23 – 0.43)

Abbreviations: AUC, area under the curve; CI, confidence interval; Intra-Op, intraoperative; LOS, length of hospital stay; LR, logistic regression; Peri-Op, perioperative; XGBoost, extreme gradient boosting.

**Table 3. -:** Importance of Different Datasets

Surgery Type	Outcome	Intra-Op	Peri-Op	Peri-Op Cognitive
**All surgeries**	LOS			√
	Charges			√
	Average Pain		√	
	1-year Mortality			√
**Orthopedics**	LOS			√
	Charges			√
	Average Pain		√	
**Neurology**	LOS	√		
	Charges			√
	Average Pain	√		
**Cardiac and vascular**	LOS		√	
	Charges			√
	Average Pain	√		
**Urology**	LOS			√
	Charges			√
	Average Pain			√
**Gynecology**	LOS			√
	Charges	√		
	Average Pain		√	
**Otolaryngology**	LOS			√
	Charges		√	
	Average Pain	√		

Abbreviations: Intra-Op, intraoperative; LOS, length of hospital stay; Peri-Op, perioperative.

## Data Availability

Datasets are available upon reasonable request. Reasonable requests will be reviewed to monitor compliance with the concerned authorities: the National Institutes of Health (NIH) and the University of Florida Institutional Review Board (IRB). Relevant clinical trial numbers for the studies from which the datasets in this study have been constructed are NCT01986577 and NCT03175302.

## Abbreviations

AdaBoost: adaptive boosting
ADI: area deprivation index
AI: artificial intelligence
ASA: American Society of Anesthesiologists
AUC: area under the curve
AUROC: area under the receiver operating curve
AVG NIBP: average deviation of noninvasive blood pressure
BMI: body mass index
CatBoost: categorical boosting
CD: cognitive disorder
CDT: clock drawing test
DB: diabetes
DL: deep learning
HCl: hydrochloride
HIPAA: Health Insurance Portability and Accountability Act
HL: hyperlipidemia
HT: hypertension
iso-sev-MAC: mean alveolar concentration of isoflurane and sevoflurane
LOS: length of hospital stay
LR: logistic regression
MD: movement disorder
ML: machine learning
MLP: multilayered perceptron
MMSE: Mini Mental State Exam
MRI: magnetic resonance imaging
MME: morphine milligram equivalent
Peri-AIIMS: perioperative artificial intelligence–based integrated modeling of surgeries
RF-VAE: relevance-factor variational autoencoder
SA: sleep apnea
SD NIBP: standard deviation of noninvasive blood pressure
SHAP: Shapley Additive Explanations
UF: University of Florida
VAE: variational autoencoder
XGBoost: extreme gradient boosting
